# β‐Endorphin Mediates Electroacupuncture‐Induced Remyelination via Neural Stem Cell Lineage Modulation in Experimental Autoimmune Encephalomyelitis

**DOI:** 10.1111/cns.70658

**Published:** 2025-11-24

**Authors:** Yanping Wang, Xiaoru Ma, Zhixin Qiao, Wei Zhuang, Xiyu Zhang, Jingyu Luo, Junfeng Wu, Anqi Li, Chao Wang, Jiayu Ji, Xin Xiu, Jing Wang, Yanting Meng, Wei Huang, Sifan Zhang, Xiujuan Lang, Xijun Liu, Bo Sun, Hulun Li, Yumei Liu

**Affiliations:** ^1^ Department of Neurobiology, School of Basic Medical Sciences Harbin Medical University Harbin Heilongjiang P.R. China; ^2^ The Key Laboratory of Preservation of Human Genetic Resources and Disease Control in China Harbin Medical University, Ministry of Education Harbin Heilongjiang P.R. China; ^3^ The Key Laboratory of Myocardial Ischemia Harbin Medical University, Ministry of Education Harbin Heilongjiang P.R. China

**Keywords:** β‐endorphin (β‐EP), electroacupuncture (EA), experimental autoimmune encephalomyelitis (EAE), neural stem cells (NSCs), oligodendrocyte progenitor cells (OPCs)

## Abstract

**Aims:**

Effective remyelination in multiple sclerosis (MS) requires both the proliferation of endogenous neural stem cells (NSCs) and their lineage‐specific differentiation into oligodendrocyte progenitor cells (OPCs). This study aimed to investigate whether electroacupuncture (EA) promoted NSC proliferation and OPC differentiation via β‐endorphin (β‐EP)–mediated opioid signaling in a murine model of MS.

**Methods:**

Experimental autoimmune encephalomyelitis (EAE) was induced in C57BL/6 mice to model MS. EA stimulation was applied daily at the Zusanli (ST36) acupoint. NSC proliferation and OPC differentiation were assessed via immunofluorescence, flow cytometry (FCM), and RT‐qPCR. β‐EP expression and opioid receptor involvement were evaluated in the hypothalamus and subventricular zone (SVZ). Naloxone, a nonselective opioid receptor antagonist, was used to determine the role of opioid signaling in EA‐induced effects.

**Results:**

EA significantly enhanced NSC proliferation and increased the proportion of NSC‐derived OPCs in the SVZ of EAE mice. EA treatment improved clinical score, reduced demyelination, and attenuated leukocyte infiltration of the central nervous system (CNS). Mechanistically, EA upregulated β‐EP and its precursor pro‐opiomelanocortin (POMC), along with opioid receptors μ‐opioid receptor (MOR) and κ‐opioid receptor (KOR) (encoded by *Oprm1* and *Oprk1*, respectively). Naloxone administration abolished the beneficial effects of EA on NSC behavior and remyelination, confirming the involvement of opioid receptor‐dependent β‐EP signaling.

**Conclusion:**

EA promotes remyelination in EAE mice by stimulating β‐EP‐mediated NSC proliferation and OPC differentiation. These findings reveal a novel neuroregenerative mechanism and support EA as a promising adjunctive strategy for demyelinating diseases such as MS.

## Graphical abstract

Electroacupuncture at ST36 enhances remyelination in EAE mice by promoting neural stem cell proliferation and OPC differentiation through β‐endorphin–mediated opioid receptor signaling. These effects are blocked by naloxone, highlighting a novel endogenous opioid‐dependent mechanism for neuroregeneration.

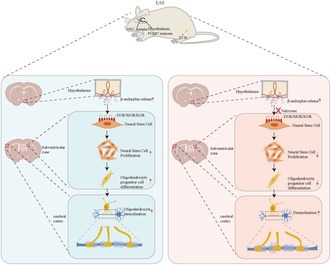

## 1 Introduction

Multiple sclerosis (MS) is a chronic demyelinating disorder of the central nervous system (CNS) primarily affecting young adults and characterized by oligodendrocyte loss and insufficient remyelination [1]. Although current immunomodulatory therapies mitigate inflammatory lesions, they fail to promote functional repair of demyelinated axons or halt progressive neurodegeneration. Recent studies highlight the potential of neural stem cells (NSCs) to contribute to remyelination by differentiating into oligodendrocyte progenitor cells (OPCs), yet this endogenous regenerative process remains inefficient in the pathological milieu of MS [2, 3]. Targeting NSCs to enhance their proliferation and OPC‐lineage differentiation is emerging as a promising reparative strategy [4, 5]. NSCs reside mainly in the subventricular zone (SVZ) and hippocampus and are activated by injury signals, yet their spontaneous activity is insufficient to drive meaningful remyelination [6, 7]. Novel therapeutic approaches are needed to stimulate this endogenous repair cascade.

Electroacupuncture (EA), a modern adaptation of traditional Chinese acupuncture combined with electrical stimulation, has been shown to modulate the neural microenvironment and facilitate stem cell survival and differentiation [8, 9, 10, 11]. However, while EA has been reported to promote neurogenesis in various CNS injury models, its ability to specifically drive NSC‐to‐OPC lineage commitment and remyelination in MS remains unexplored.

The endogenous opioid system, such as opioid peptide hormone β‐EP and μ‐opioid receptor (MOR), κ‐opioid receptor (KOR), and δ‐opioid receptor (DOR) (encoded by *Oprm1*, *Oprk1* and *Oprd1*, respectively), is widely distributed in the CNS, plays roles as neuromodulators and neurotransmitters, and is closely related to the immune system [12]. More importantly, the endogenous opioid system, particularly the β‐endorphin (β‐EP) signaling axis, has recently emerged as a neuromodulatory regulator of neurogenesis [13, 14, 15]. Yet, whether β‐EP mediates the effect of EA on NSC lineage decisions, especially toward OPCs during demyelinating disease, remains unknown. Given that opioid receptors are widely expressed in NSCs and glial precursors, investigating their role in EA‐induced neurorepair may reveal a novel signaling mechanism for regenerative modulation [12].

In this study, we hypothesized that EA at the ST36 acupoint promoted the proliferation and OPC differentiation of endogenous NSCs via β‐EP signaling, leading to enhanced remyelination in the experimental autoimmune encephalomyelitis (EAE) model. By integrating neuroimmunological, cellular, and pharmacological approaches, we aimed to uncover a previously uncharacterized link between EA stimulation and opioid‐modulated NSC differentiation. These findings could offer a new mechanistic rationale for EA as a complementary strategy to promote CNS repair in MS.

## 2 Results

### 2.1 EA at ST36 Promoted the Proliferation of NSCs in EAE Model Mice

**FIGURE 1:**
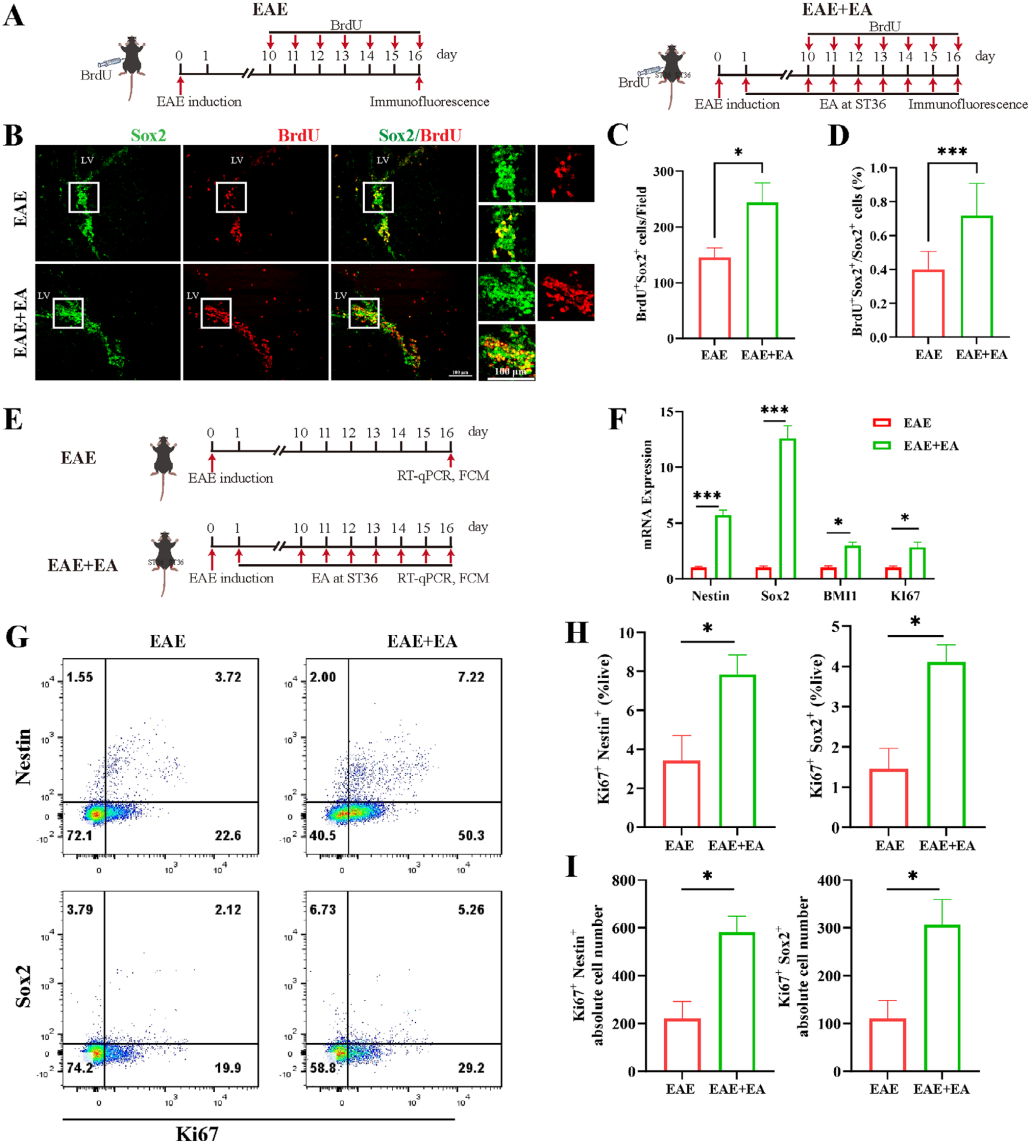
EA at ST36 promoted the proliferation of NSCs in the EAE model mice. (A) Schematic diagram of the procedure for treating EAE with BrdU and EA at ST36 for immunofluorescence staining. (B) Representative images of BrdU^+^ Sox2^+^ NSCs in the SVZ. Scale bar, 100 μm. (C) Statistical analysis of the number of BrdU^+^ Sox2^+^ NSCs in the field. (D) Statistical analysis of the ratio of BrdU^+^ Sox2^+^ NSCs in Sox2^+^ cells in the field. (E) Schematic diagram of the procedure for treating EAE with EA at ST36 for RT‐qPCR and FCM. (F) The gene expression of *Nestin*, *Sox2*, *Bmi1*, and *Ki67* in the SVZ. (G) Representative flow scatter plots of Ki67^+^ Nestin^+^ NSCs and Ki67^+^ Sox2^+^ NSCs. (H) Statistical analysis of the ratio of Ki67^+^ Nestin^+^ NSCs and Ki67^+^ Sox2^+^ NSCs. (I) Statistical analysis of the absolute number of Ki67^+^ Nestin^+^ NSCs and Ki67^+^ Sox2^+^ NSCs. The data were presented as the means ± SEM and analyzed via one‐way ANOVA followed by Tukey's post hoc test (F) and unpaired *t*‐test (C, D, H, I). *n* = 3 or 4. *p < 0.05, ***p < 0.001.

Since NSCs can differentiate into a variety of cells to perform functions related to regeneration, we investigated the effect of EA at ST36 on NSC proliferation. After the successful establishment of the EAE disease model, BrdU (80 mg/kg) was injected intraperitoneally once daily from day 10 to day 16 after EAE induction to trace proliferating NSCs, and the samples were collected on day 16 for immunofluorescence staining (Figure 1A). The results revealed that EA at ST36 significantly increased the proportion and absolute number of BrdU^+^ Sox2^+^ NSCs in the SVZ (Figure 1B–D). The levels of the NSC markers Nestin and Sox2, the proliferation marker Bmi1, and Ki67 in the brain were also assessed on day 16 by RT‐qPCR and FCM (Figure 1E). EA at ST36 obviously upregulated the gene expression levels of *Nestin*, *Sox2*, *Bmi1*, and *Ki67* in the SVZ (Figure 1F). The FCM results revealed that EA at ST36 significantly promoted the proliferation of Nestin^+^ NSCs and Sox2^+^ NSCs, and both the proportion and absolute number of Ki67^+^ Nestin^+^ NSCs and Ki67^+^ Sox2^+^ NSCs were increased (Figure 1G–I). Taken together, these results indicated that EA at ST36 promoted the proliferation of NSCs.

### 2.2 EA at ST36 Promoted the OPC Differentiation of NSCs and the Remyelination of Oligodendrocytes in EAE Model Mice

**FIGURE 2:**
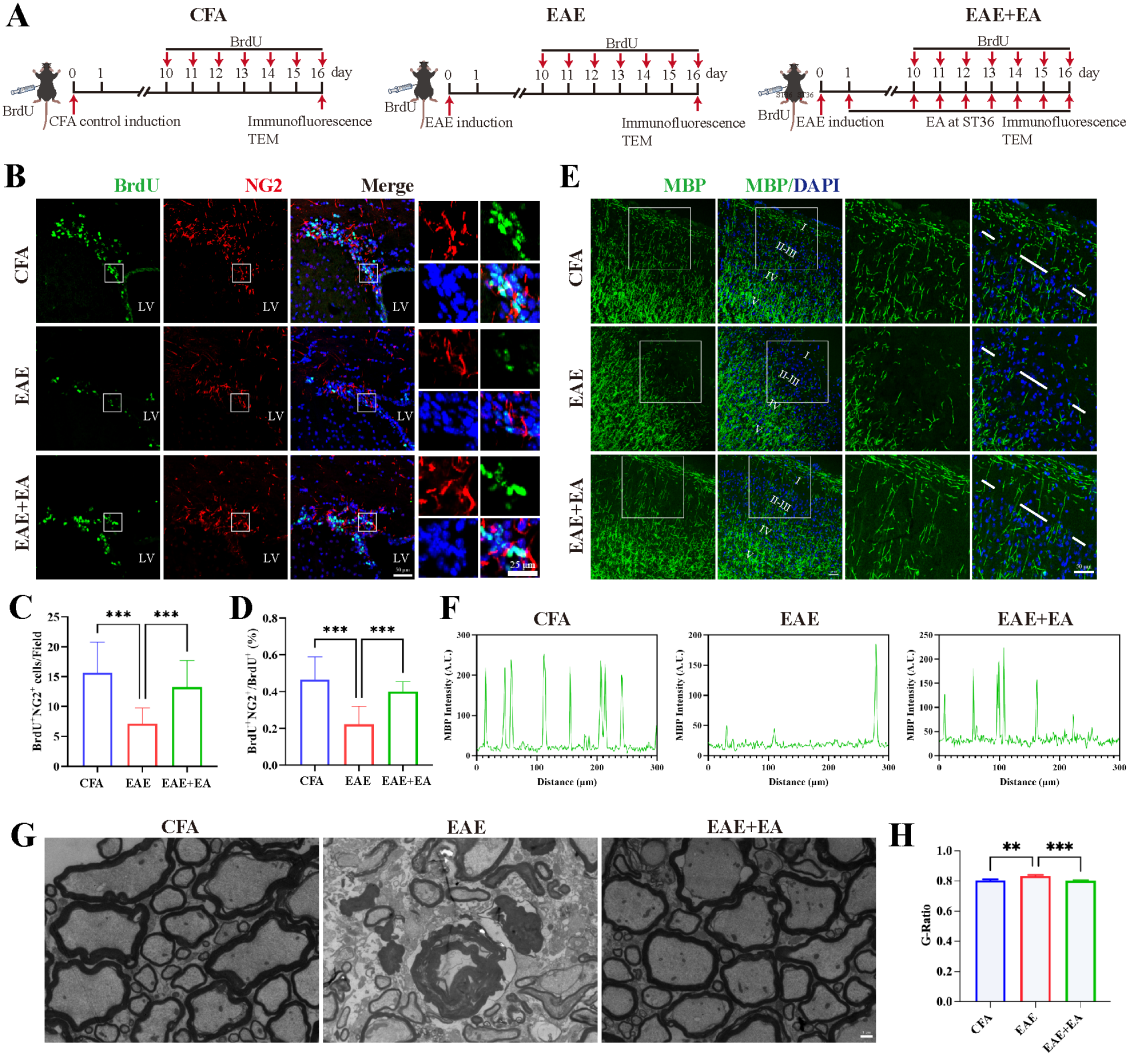
EA at ST36 promoted the OPC differentiation of NSC and the remyelination of oligodendrocytes in EAE model mice. (A) Schematic diagram of the treatment of model mice with BrdU and EA at ST36 for immunofluorescence staining and TEM. (B) Representative images of BrdU^+^ NG2^+^ OPCs in the SVZ. Scale bar, 50 and 25 μm. (C) Statistical analysis of the number of BrdU^+^ NG2^+^ OPCs in the field. (D) Statistical analysis of the ratio of BrdU^+^ NG2^+^ OPCs in BrdU^+^ cells in the field. (E) Representative images of MBP^+^ cells in the cerebral cortex. The right column is a zoomed region from the left column. Scale bar, 50 μm. (F) MBP intensity at layers II‐III in the CFA control group, EAE group and EAE + EA group. (G) Representative TEM images showing the ultrastructure of myelin sheaths. Scale bar: 1 μm. (H) G‐ratio of myelinated fiber in G (*n* ≥ 100). The data were presented as the means ± SEM and analyzed via one‐way ANOVA followed by Tukey's post hoc test (C, D, H). *n* = 3. **p < 0.01, ***p < 0.001.

During the demyelination process, OPCs were mobilized to the demyelinated sites and subsequently differentiated into mature oligodendrocytes to remyelinate the damaged axons. NSCs can differentiate into a variety of cells to perform functions related to regeneration. Moreover, the studies described above revealed that EA at ST36 promoted the proliferation of NSCs in the SVZ. Therefore, we subsequently investigated the effect of EA at ST36 on NSC differentiation. The samples were collected on day 16 after EAE induction (Figure 2A). The results of immunofluorescence staining revealed that EA at ST36 significantly increased the proportion and the absolute number of BrdU^+^ NG2^+^ OPCs in the SVZ (Figure 2B–D). MBP staining was substantially reduced in EAE model mice, indicating severe demyelination, whereas in the EAE + EA group, demyelination was significantly mitigated (Figure 2E,F). TEM results revealed that compared with the EAE group, the myelin sheath in the EAE + EA group was obviously thicker (Figure 2G,H). These findings showed that EA at ST36 promoted the OPC differentiation of NSC and remyelination of oligodendrocytes in EAE model mice.

### 2.3 EA at ST36 Upregulated the Expression of POMC and β‐EP in EAE Model Mice

**FIGURE 3:**
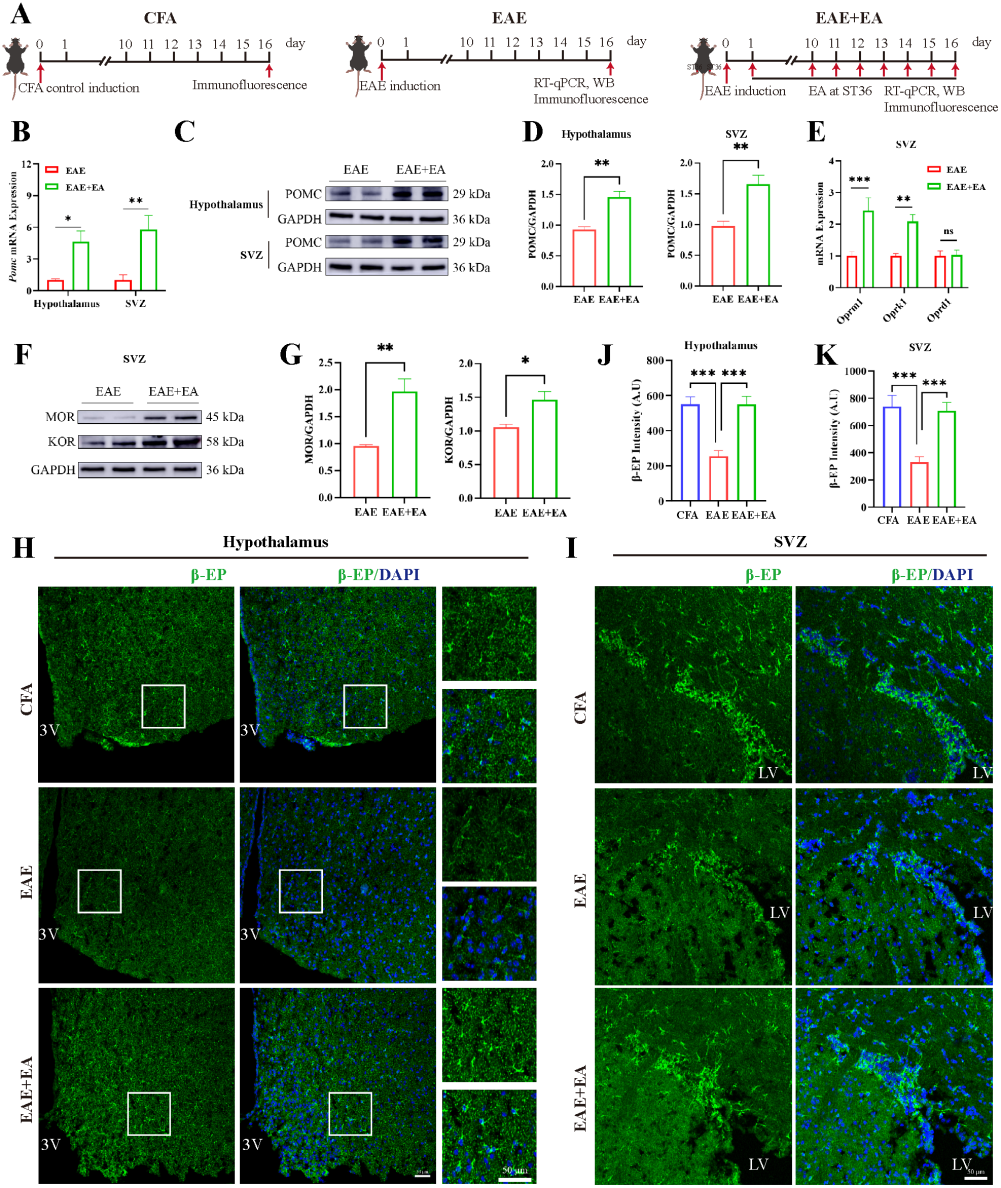
EA at ST36 upregulated the expression of POMC and β‐EP in EAE model mice. (A) Schematic diagram of the procedure for treating model mice for RT‐qPCR, WB and immunofluorescence staining. (B) Gene expression of *Pomc* in the hypothalamus and SVZ. (C) WB analysis of the expression of POMC in the hypothalamus and SVZ. (D) The statistical results of POMC expression in (C). (E) Gene expression of the opioid receptors *Oprm1*, *Oprk1* and *Oprd1* in SVZ. (F) WB analysis of the expression of MOR and KOR in SVZ. (G) The statistical results of MOR and KOR expression in (F). (H) Representative images of β‐EP in the hypothalamus. Scale bar, 50 μm. (I) Representative images of β‐EP in SVZ. Scale bar, 50 μm. (J) Statistical analysis of the β‐EP intensity in the hypothalamus. (K) Statistical analysis of the β‐EP intensity in SVZ. The data were presented as the means ± SEM and analyzed via one‐way ANOVA followed by Tukey's post hoc test (B, E, J, K) and unpaired *t*‐test (D, G). *n* = 3 or 4.*p < 0.05, **p < 0.01, ***p < 0.001.

As mentioned above, pro‐opiomelanocortin (POMC) neurons regulate the proliferation and neuronal differentiation of NSCs in the SVZ through long‐range projections to promote the repair of injury. The endogenous opioid system has been suggested to play a role in the pathogenesis of MS. Therefore, we measured the expression of POMC, β‐EP and opioid receptors in the hypothalamus and SVZ (Figure 3A). The RT‐qPCR and WB results revealed that EA at ST36 significantly upregulated the expression of POMC in the hypothalamus and SVZ (Figure 3B–D). The gene expression of the opioid receptors *Oprm1* and *Oprk1* was upregulated in SVZ after EA at ST36 (Figure 3E). WB further revealed that EA at ST36 significantly upregulated the protein level of MOR and KOR in SVZ (Figure 3F,G). Moreover, immunofluorescence staining revealed that EA at ST36 increased β‐EP expression in the hypothalamus and SVZ (Figure 3H–K). The above results revealed that EA at ST36 upregulated POMC and β‐EP in the hypothalamus and SVZ in the treatment of EAE. Therefore, in the subsequent experiment, we experimentally assessed the effect of an opioid receptor antagonist.

### 2.4 Naloxone Attenuated the Therapeutic Effect of EA at ST36 in EAE Model Mice

**FIGURE 4:**
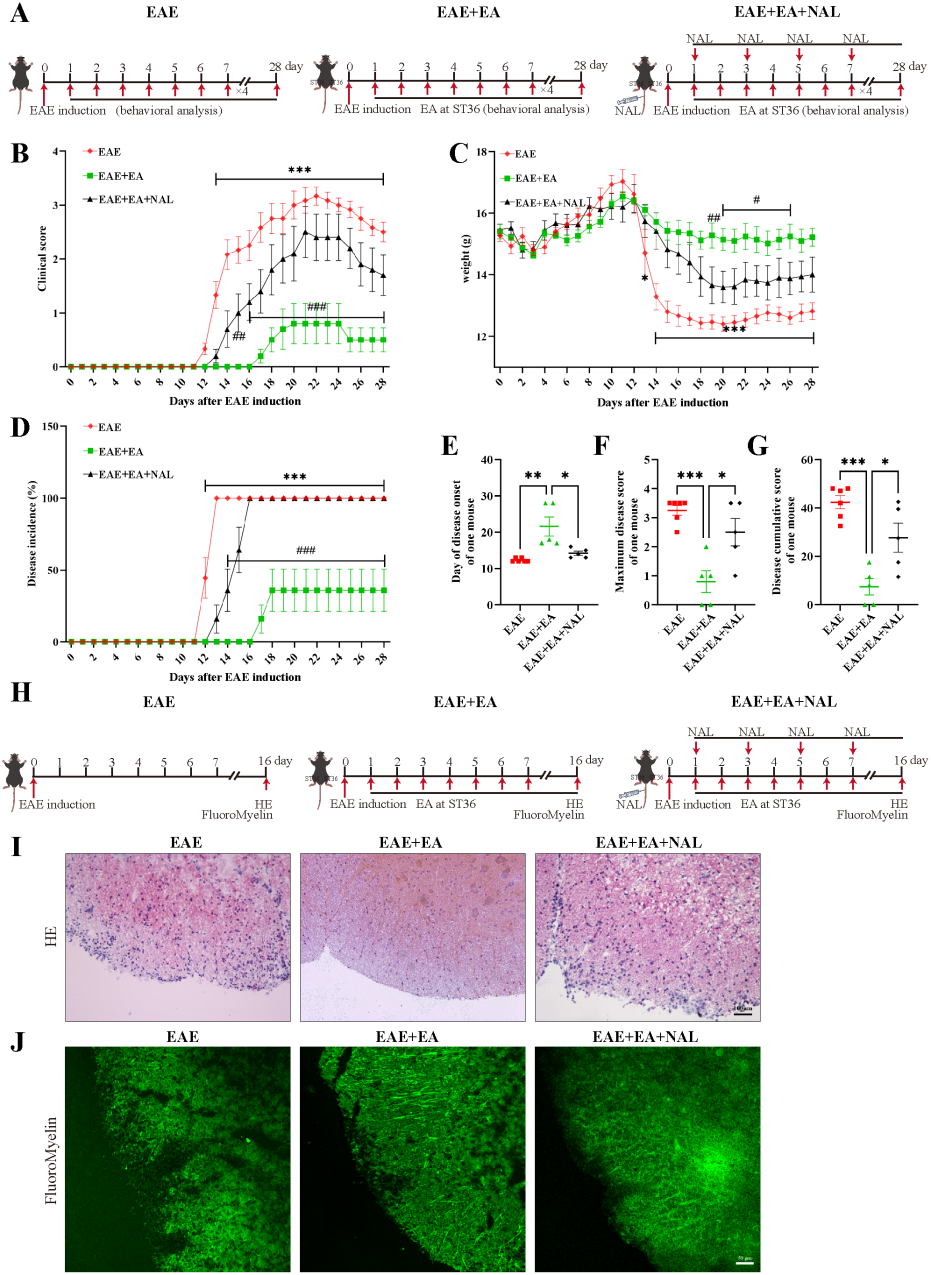
Naloxone attenuated the therapeutic effect of EA at ST36 in EAE model mice. (A) Schematic diagram of the procedure for treating EAE with EA at ST36 and NAL. (B) The clinical score of three groups. (C) The weight loss of three groups. (D) The disease incidence for showing the clinical symptoms of EAE in three groups. (E) The day of disease onset of one mouse. (F) The maximum disease score of one mouse. (G) The disease cumulative score of one mouse. (H) Schematic diagram of the procedure for treating EAE with EA at ST36 and NAL for HE and FluoroMyelin green staining. (I) Representative images of leukocyte infiltration in the lumbar spinal cord, scale bar, 100 μm. (J) The demyelination of EAE in the spinal cord, scale bar, 50 μm. The data were presented as the means ± SEM and analyzed via two‐way ANOVA followed by Tukey post hoc test (B–D) and one‐way ANOVA coupled with Tukey post hoc test (E–G). *n* = 5 or 6. (B–D) *EAE group vs. EAE + EA group, ^#^EAE + EA group vs. EAE + EA + NAL group. *p< 0.05, **p < 0.01, ***p < 0.001. ^#^ *p* < 0.05, ^##^ *p* < 0.01, ^###^ *p* < 0.001.

EA at ST36 upregulated the expression of POMC, β‐EP, and opioid receptors in the hypothalamus and SVZ of EAE model mice. We next assessed the effect of the nonselective opioid receptor antagonist naloxone (NAL) on EA at ST36 in the treatment of EAE (Figure 4A). Compared with the EAE group, both the EAE + EA group and the EAE + EA + NAL group presented a delayed onset of the disease, but the EAE + EA group presented a significantly delayed onset of disease on day 17 after EAE induction compared with the EAE + EA + NAL group on day 13 after EAE induction. Both the EAE + EA group and the EAE + EA + NAL group reduced clinical score. However, the EAE + EA group had lower clinical score, only showing mild tail paralysis compared with the EAE + EA + NAL group, manifesting hind limb paralysis (Figure 4B,E). Moreover, the loss of weight of the mice in the EAE + EA group was lower than that of the mice in the EAE group and EAE + EA + NAL group (Figure 4C). Further analysis revealed that the incidence rates, refering to the proportion of mice showing the clinical symptoms of EAE, were significantly different between the EAE + EA group and the EAE + EA + NAL group from day 14 after EAE induction (Figure 4D). The mitigation of the maximum clinical score and the cumulative clinical score of EA at ST36 disappeared after the application of naloxone (Figure 4F,G). Histological examination of the spinal cords was performed on day 16 after EAE induction (Figure 4H). Leukocyte infiltration could be observed in the spinal cord of the EAE group. The EAE + EA group exhibited significantly decreased leukocyte infiltration, but this effect was reversed by naloxone in the EAE + EA + NAL group (Figure 4I). Moreover, the alleviation of demyelination in the EAE + EA group was abolished after the application of naloxone (Figure 4J). The above results indicated that the therapeutic effect of EA at ST36 was attenuated by naloxone, indicating that EA at ST36 exerted a therapeutic effect on EAE via β‐EP signaling.

### 2.5 Naloxone Attenuated the Proliferation of NSCs Induced by EA at ST36 in EAE Model Mice

**FIGURE 5:**
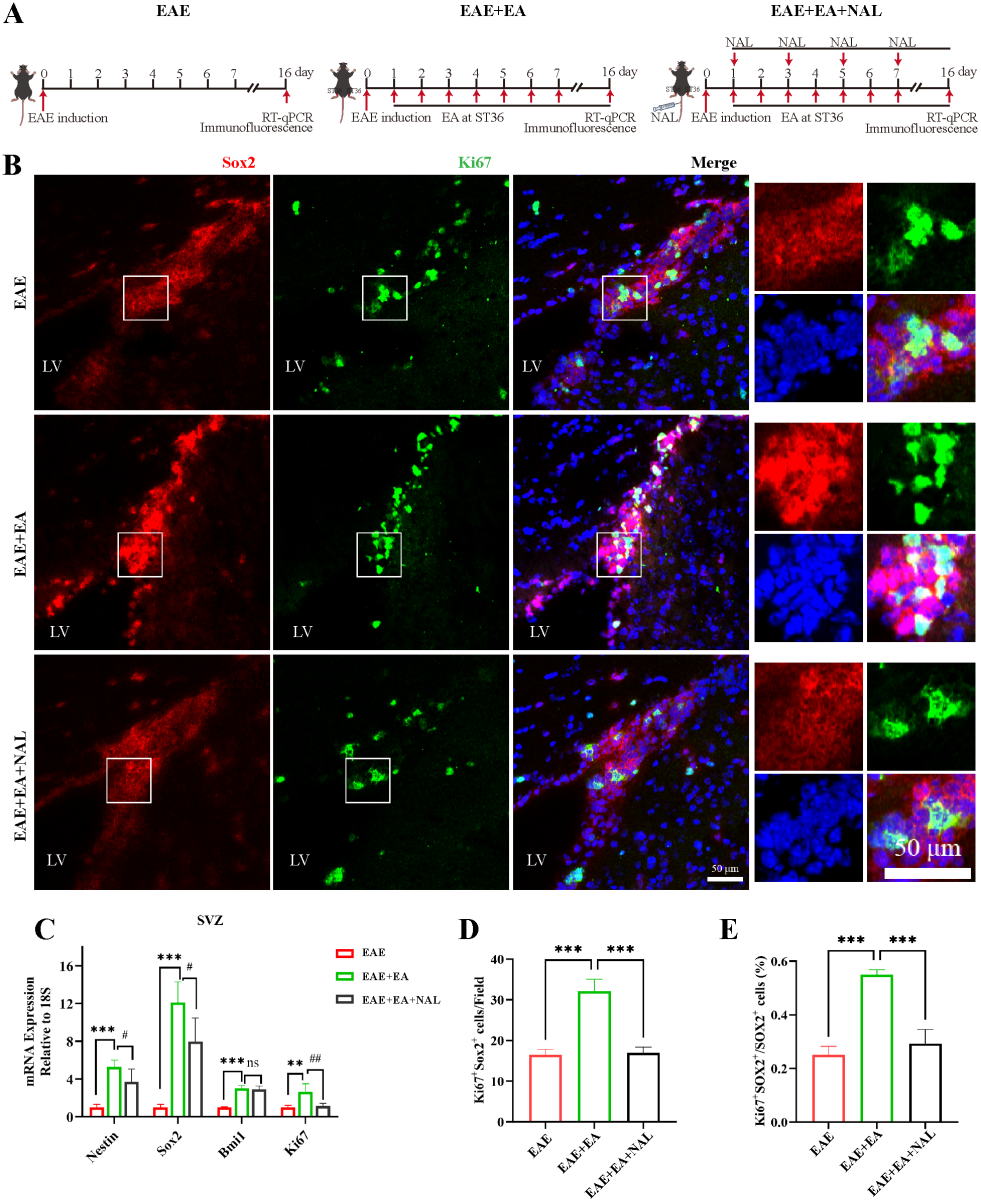
Naloxone attenuated the proliferation of NSCs induced by EA at ST36 in the EAE model mice. (A) Schematic diagram of the procedure for treating EAE with NAL for RT‐qPCR and immunofluorescence staining. (B) Representative images of Ki67^+^ Sox2^+^ NSCs in the SVZ. Scale bar, 50 μm. (C) The gene expression of *Nestin*, *Sox2*, *Bmi1*, and *Ki67* in the SVZ. (D) Statistical analysis of the number of Ki67^+^ Sox2^+^ NSCs in the field. (E) Statistical analysis of the ratio of Ki67^+^ Sox2^+^ NSCs in Sox2^+^ cells in the field. The data were presented as the means ± SEM and analyzed via one‐way ANOVA followed by Tukey's post hoc test. *n* = 3 or 4. (C) *EAE group vs. EAE + EA group, ^#^EAE + EA group vs. EAE + EA + NAL group. **p < 0.01 ***p < 0.001. ^#^ *p* < 0.05, ^##^ *p* < 0.01.

We next explored the effect of the proliferation of NSCs by EA at ST36 after the application of naloxone (Figure 5A). The results revealed that the upregulation of *Nestin*, *Sox2*, *Bmi1*, and *Ki67* gene expression in the SVZ of the EAE + EA group was abolished after the application of naloxone except for *Bmi1* (Figure 5C). Similarly, immunofluorescence staining revealed that after the application of naloxone, the increase in the proportion and absolute number of Ki67^+^ Sox2^+^ NSCs in the SVZ by EA at ST36 was significantly attenuated (Figure 5B,D,E). The above results indicated that after the application of naloxone, EA at ST36 could not effectively promote the proliferation of NSCs in the SVZ of EAE model mice.

### 2.6 Naloxone Attenuated the OPC Differentiation of NSCs and Oligodendrocyte Remyelination Induced by EA at ST36 in EAE Model Mice

**FIGURE 6:**
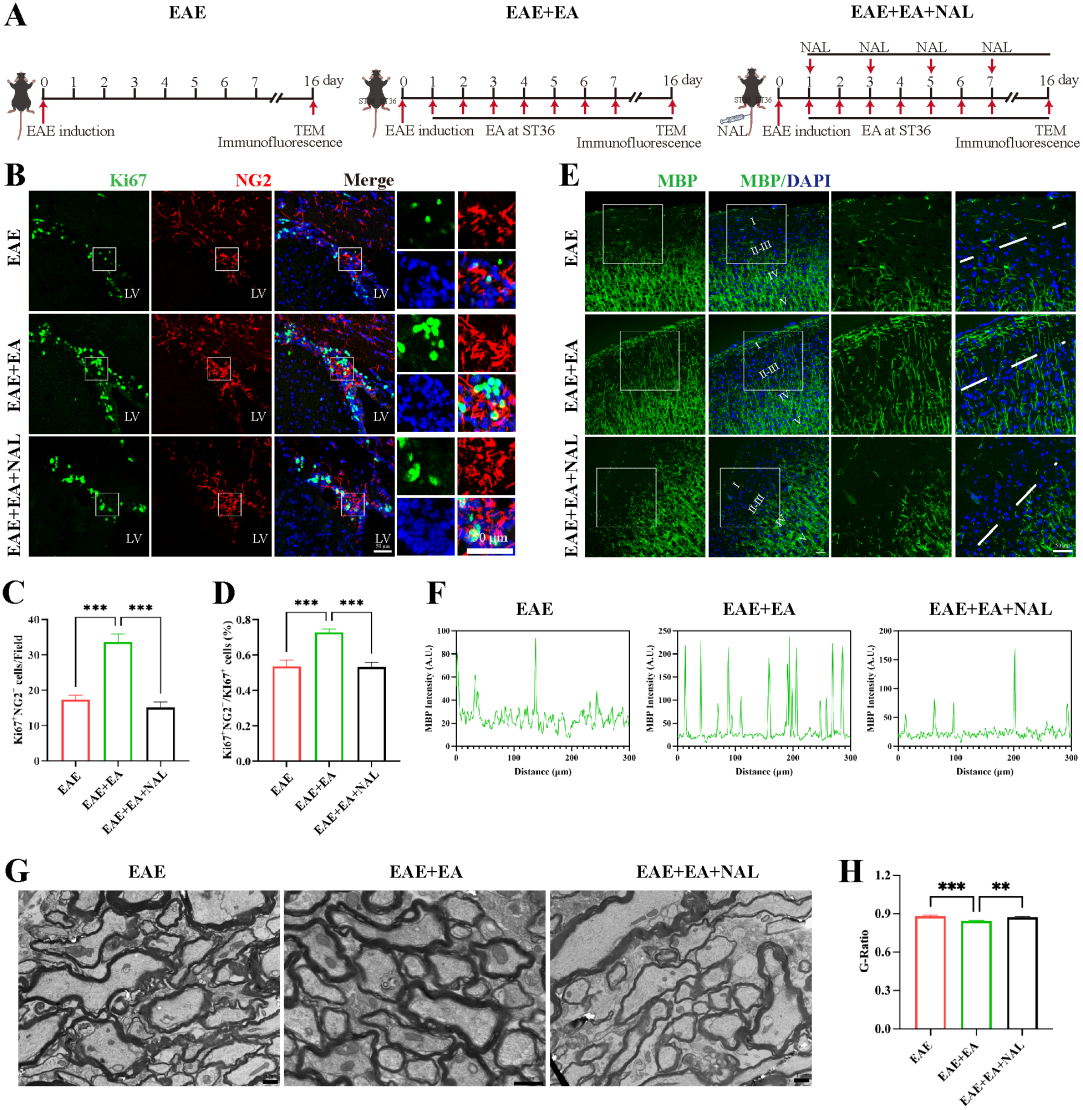
Naloxone attenuated the OPC differentiation of NSCs and oligodendrocyte remyelination induced by EA at ST36 in EAE model mice. (A) Schematic diagram of the procedure for treating EAE with EA at ST36 and NAL for TEM and immunofluorescence staining. (B) Representative images of Ki67^+^ NG2^+^ OPCs in the SVZ. Scale bar, 50 μm. (C) Statistical analysis of the number of Ki67^+^ NG2^+^ OPCs in the field. (D) Statistical analysis of the ratio of Ki67^+^ NG2^+^ OPCs in Ki67^+^ cells in the field. (E) Representative images of MBP^+^ cells in the cerebral cortex. The right column is a zoomed region from the left column. Scale bar, 50 μm. (F) MBP intensity at layers II‐III in the EAE group, EAE + EA group and EAE + EA + NAL group. (G) Representative TEM images showing the ultrastructure of myelin sheaths. Scale bar: 1 μm. (H) G‐ratio of myelinated fiber in G (*n* ≥ 100). The data were presented as the means ± SEM and analyzed via one‐way ANOVA followed by Tukey's post hoc test. *n* = 3. **p < 0.01, ***p < 0.001.

The above results indicated that naloxone attenuated the proliferation of NSCs induced by EA at ST36 in EAE model mice. Next, we observed the effect of naloxone on the OPC differentiation of NSCs by EA at ST36 in the EAE model mice (Figure 6A). Immunofluorescence staining revealed that EA at ST36 significantly increased the proportion and absolute number of BrdU^+^ NG2^+^ OPCs in the SVZ as mentioned previously (Figure 2B–D). Ki67^+^ NG2^+^ OPCs were also detected in the present study. Immunofluorescence staining revealed that the absolute number and proportion of Ki67^+^ NG2^+^ OPCs were increased after EA at ST36, but naloxone attenuated the OPC differentiation of NSCs induced by EA at ST36 in EAE model mice (Figure 6B–D). Oligodendrocytes in the cerebral cortex were identified by immunofluorescence staining. After the application of naloxone, MBP staining in the cerebral cortex in the EAE + EA + NAL group was significantly reduced (Figure 6E,F). TEM results revealed that compared with the EAE group, the myelin sheath in the EAE + EA group was obviously thicker, which was obviously thinner in the EAE + EA + NAL group than that in the EAE + EA group (Figure 6G,H). These findings suggested that naloxone attenuated the OPC differentiation of NSCs and oligodendrocyte remyelination. EA at ST36 alleviated EAE and promoted the proliferation and OPC differentiation of NSCs in the SVZ and oligodendrocyte remyelination in EAE model mice via opioid receptor‐mediated β‐EP signaling.

## 3 Discussion

In the treatment of CNS degenerative diseases, such as MS [16, 17], complementary and alternative medicine (CAM), including acupuncture and EA, has gained increasing attention for its neuroprotective properties and minimal side effects. MS, characterized by chronic inflammation and oligodendrocyte demyelination, currently lacks therapies that effectively promote remyelination [18, 19]. Given that remyelination failure in MS is largely attributed to a blockade in OPC differentiation, strategies that target this process are of considerable interest.

Previous studies have demonstrated that EA can promote endogenous NSC proliferation and OPC survival in various CNS injury models [20, 21, 22]. However, the molecular mechanisms underlying EA‐induced lineage differentiation of NSCs into OPCs, particularly in the context of MS, remain largely unexplored. This study aimed to bridge this gap by investigating the role of β‐EP and opioid receptor signaling in EA‐mediated regulation of NSC fate and remyelination.

Our study demonstrated for the first time that EA at ST36 significantly promoted NSC proliferation and their differentiation into OPCs in the SVZ of EAE mice. This effect was accompanied by a reduction in leukocyte infiltration, an improved clinical score, and enhanced remyelination. Mechanistically, EA markedly increased β‐EP expression in the hypothalamus and SVZ, and this neuroprotective effect was abolished by naloxone, indicating that EA‐induced remyelination and NSC‐OPC lineage progression were mediated through opioid receptor‐dependent β‐EP signaling. Notably, the identification of β‐EP as a central mediator of the effects of EA on NSC fate commitment represented a novel mechanistic insight. While opioid signaling had been implicated in neurogenesis, its role in regulating NSC‐to‐OPC differentiation in demyelinating disease models had not been clearly elucidated prior to this study. Our results expanded the understanding of endogenous opioid pathways in neuroregeneration and highlighted EA as a modulator of this process.

Studies have revealed that opioid ligands and their receptors are involved in the pathological processes of MS and that endogenous opioid peptide expression levels are lower in MS patients and EAE animal models than in healthy controls [23, 24]. Our current results also showed that EA upregulated the expression of the endogenous opioid peptide β‐EP. The expression of POMC and β‐EP was downregulated in the EAE group compared with the CFA control group; however, EA at ST36 upregulated POMC and β‐EP in the hypothalamus and SVZ in the treatment of EAE. Previous literature has reported conflicting roles of opioid receptors in NSC and OPC differentiation, with studies showing both promotion and inhibition depending on the receptor subtype, ligand source, and disease context [25, 26, 27, 28]. Our study aligns with reports that activation of KOR [25] and MOR [26] enhances oligodendrogenesis and remyelination. However, further mechanistic studies are warranted to differentiate the contributions of various POMC‐derived peptides, such as ACTH and α‐/β‐/γ‐MSH, which may also be modulated by EA.

Several limitations should be acknowledged. First, our findings are based on the EAE mouse model, which does not fully replicate human MS pathology. Second, β‐EP is only one of several POMC‐derived peptides, and its isolated effects require validation using β‐EP knockout or receptor‐specific blockade models. Third, while naloxone abolishes EA‐induced effects, opioid receptor subtype‐specific involvement remains to be clarified. Finally, the long‐term effects of EA on remyelination and disease progression remain to be explored. Despite these limitations, our study offers a foundational framework for understanding how EA modulates endogenous repair processes via β‐EP signaling. These insights provide a rationale for clinical translation and justify future investigations into the therapeutic utility of EA in remyelination strategies for MS. Moreover, while EA is generally regarded as very safe compared to pharmacological treatments, possible side effects may include minor local reactions (e.g., bruising, pain at insertion sites) and rare but serious complications (e.g., pneumothorax, infections, or nerve injury) [29, 30]. These risks are largely avoidable when EA is performed by trained professionals under proper standards. Overall, EA remains a safe and well‐tolerated complementary therapy.

In conclusion, this study reveals a previously uncharacterized β‐EP‐dependent mechanism by which EA promotes NSC proliferation, OPC differentiation, and remyelination in EAE mice. These findings position EA not only as a neuroprotective intervention but also as a potential regenerative strategy that warrants further exploration in clinical settings. Bridging traditional medicine with modern neurobiology, this work paves the way for integrating EA into evidence‐based therapies for demyelinating diseases.

## 4 Methods

### 4.1 Animals

The 129 mice used in this study were 6–8‐week‐old female C57BL/6J mice purchased from Beijing HFK Bioscience Co. Ltd. (Beijing, China) and weighing 16–18 g. The mice received food and water ad libitum. They were housed at a temperature of 21°C ± 2°C and relative humidity of 50% ± 5% on a 12 h light/dark cycle under specific pathogen‐free conditions. The Ethics Committee of Harbin Medical University approved all animal experiments. The work has been reported in line with the ARRIVE guidelines 2.0.

### 4.2 Grouping

This study was divided into two parts. Randomization was employed to allocate the mice to control and treatment groups. First, we explored the effects of EA treatment on the proliferation and OPC differentiation of NSCs in mice with EAE. The mice were divided into three groups: the CFA control group (CFA), the EAE group (EAE), and the EAE with EA treatment group (EAE + EA), comprising 25 mice per subgroup. Second, we aimed to clarify the underlying mechanism by which EA treatment affects the proliferation and OPC differentiation of NSCs in mice with EAE. The mice were divided into three groups: the EAE group (EAE), the EAE with EA treatment group (EAE + EA), and the EAE and EA treatment combined with naloxone injection group (EAE + EA + NAL), comprising 18 mice per subgroup. EA was applied from the first day after EAE induction (2 Hz, 30 min/day). EA stimulation was inserted into the bilateral ST36 acupoint at a depth of 3 mm and attached to an EA instrument (Model G6805; Shanghai Marine Instrument General Factory, Shanghai, China). Mice in the EAE + EA + NAL group were intravenously injected with 4 mg/kg naloxone (357‐08‐4; Tocris, Bristol, UK) via the tail vein 30 min before EA treatment every other day. To minimize potential confounding factors, the following strategies were adopted: the treatment sequence for each group was randomly arranged. Moreover, the way of animal rearing was designed to balance the cage usage among different treatment groups, in order to avoid positional deviations.

### 4.3 EAE Animal Model Establishment

In this study, we constructed an EAE animal model as previously described. In brief, 100 μL of an emulsion of complete Freund's adjuvant (CFA) (Sigma‐Aldrich, St. Louis, MO, USA) containing 250 μg of *mycobacterium tuberculosis* strain H37Ra (BD Biosciences, San Jose, CA, USA) and 200 μg of myelin oligodendrocyte glycoprotein peptide 35–55 (MOG_35–55_) (MEVGWYRSPFSRVVHLYRNGK) (Bioyear Gene Biotechnology Inc., Wuhan, China) was subcutaneously injected into two sites of the bilateral axilla of the mice. 200 ng pertussis toxin (List Biological Laboratories Inc., Campbell, CA, USA) was intravenously injected into the tail vein on days 0 and 2 after EAE induction. In the CFA control group, phosphate‐buffered saline (PBS) was used instead of MOG_35–55_ to prepare the immune emulsion, and pertussis toxin was not administered.

The clinical scores of the mice were assessed daily. The severity of EAE was graded according to the following criteria: 0, normal; 1, tail paralysis; 2, hind limb weakness or impaired gait; 3, complete hind limb paralysis; 4, complete forelimb paralysis and hind limb paralysis; and 5, moribund condition or death. When the clinical signs were intermediate between the two disease grades, 0.5 was added to the lower score. All experiments were conducted using a blinded approach, ensuring that observers were unaware of the group assignments of the mice.

### 4.4 5‐Bromo‐2′‐Deoxyuridine (BrdU) Injection

5‐Bromo‐2′‐deoxyuridine (BrdU) (ab142567; Abcam, Cambridge, MA, USA) powder was dissolved in sterile PBS in the dark. BrdU (80 mg/kg) was injected intraperitoneally into experimental animals in each group daily for 7 days beginning 10 days after EAE induction.

### 4.5 Histological Analysis

The mice were anesthetized and euthanized with 2% sodium pentobarbital solution and then transcardially perfused with PBS and 4% paraformaldehyde. The brain and spinal cord tissues were removed and embedded in OCT. Then eight‐micrometer coronal sections were cut with a rotary microtome (Microm HM560 cryostat; Thermo Fisher Scientific, Waltham, MA, USA). Hematoxylin and eosin (HE) staining was performed to examine leukocyte infiltration. The severity of demyelination in the spinal cord was examined by FluoroMyelinTM green staining. For the immunofluorescence staining, after being fixed with cold acetone, the sections were pretreated with 2 M HCL for 30 min at 37°C, neutralized with 0.1 M sodium borate buffer (pH 8.5) for 10 min and blocked with 10% horse serum containing 0.3% Triton‐100 for 90 min at room temperature (RT). The primary antibodies used were rat anti‐mouse BrdU (ab6326; Abcam, Cambridge, MA, USA), rabbit anti‐mouse Sox2 (23064S; Cell Signaling Technology, Danvers, MA, USA), rat anti‐mouse Ki67 (14‐5698‐82; Thermo Fisher Scientific, Waltham, MA, USA), rabbit anti‐mouse NG2 (DF12589; Affinity Biosciences, USA), goat anti‐mouse β‐endorphin (sc‐18,264; Santa Cruz Biotechnology, USA), and rat anti‐mouse myelin basic protein (ab7349; Abcam, Cambridge, MA, USA) antibodies, which were subsequently diluted with 10% horse serum and added to the sections for 16 h at 4°C. The next day, the secondary antibodies tetramethylrhodamine‐5‐(and 6)‐isothiocyanate (TRITC) (Thermo Fisher Scientific, Waltham, MA, USA) and fluorescein isothiocyanate (FITC)‐conjugated secondary antibody (Thermo Fisher Scientific, Waltham, MA, USA) diluted with 10% horse serum were added to the sections for 60 min at RT. The nuclei were counterstained with 4′,6‐diamidino‐2‐phenylindole (DAPI). The images were captured with a fluorescence microscope (Axio Observer; Carl Zeiss, Jena, Germany). Image analyses were conducted via ImageJ (NIH, Bethesda, MD, USA).

### 4.6 Quantitative Real‐Time Polymerase Chain Reaction (RT‐qPCR) Analyses

According to the stereotaxic map of the mouse brain, the hypothalamus, and SVZ of the mouse were removed. Tissue RNA was isolated with RNAiso Plus (TaKaRa, Tokyo, Japan) according to the manufacturer's instructions. The RNA concentration was subsequently measured via a Nanodrop 2000 (Thermo Fisher Scientific, Waltham, MA, USA). Total RNA (1000 ng) was reverse transcribed with an S1000 Touch Thermal Cycler (Bio‐Rad, Hercules, CA, USA). The 10 μL reaction system contained RNA, UltraPure distilled water (Invitrogen, Carlsbad, CA, USA), 1 μL of random primer (TransGen, Beijing, China), 2 μL of M‐MLV buffer (5×) (TaKaRa, Tokyo, Japan), 0.5 μL of dNTPs (TaKaRa, Tokyo, Japan), and 0.5 μL of M‐MLV (TaKaRa, Tokyo, Japan). The cDNA was subsequently amplified with a C1000 Touch Thermal Cycler (Bio‐Rad, Hercules, CA, USA). The 10 μL reaction system contained 4 μL of cDNA, 5 μL of 2× SYBR Green qPCR Master Mix (TransGen, Beijing, China), and 1 μL of primer. The ΔΔ*C*_t_ method was used to quantify relative mRNA expression, and the values were normalized to 18S rRNA mRNA expression. The sequences of the primers used were obtained from the Primer Bank and NCBI online primer design tool primer‐blast, and the primers were synthesized by Beijing Genomics Institute (Beijing, China).

The primers used for RT‐qPCR were as follows:

– mSox2 forward 5′‐CATCCACTTCTACCCCACCTT‐3′
– mSox2 reverse 5′‐AGCTCCCTGTCAGGTCCTT‐3′
– mNestin forward 5′‐AGAGTCAGATCGCTCAGATCC‐3′
– mNestin reverse 5′‐GCAGAGTCCTGTATGTAGCCAC‐3′
– mKi67 forward 5′‐ATCATTGACCGCTCCTTTAGGT‐3′
– mKi67 reverse 5′‐GCTCGCCTTGATGGTTCCT‐3′
– mBmi1 forward 5′‐ATCCCCACTTAATGTGTGTCCT‐3′
– mBmi1 reverse 5′‐CTTGCTGGTCTCCAAGTAACG‐3′
– mPomc forward 5′‐ATGCCGAGATTCTGCTACAGT‐3′
– mPomc reverse 5′‐TCCAGCGAGAGGTCGAGTTT‐3′
– mOprk1 forward 5′‐TCCCCAACTGGGCAGAATC‐3′
– mOprk1 reverse 5′‐GACAGCGGTGATGATAACAGG‐3′
– mOprm1 forward 5′‐CAGGGCTTGTCCTTGTAAGAAA‐3′
– mOprm1 reverse 5′‐GACTCGGTAGGCTGTAACTGA‐3′
– mOprd1 forward 5′‐CCATCACCGCGCTCTACTC‐3′
– mOprd1 reverse 5′‐GTACTTGGCGCTCTGGAAGG‐3′
– m18s forward 5′‐AGTCCCTGCCCTTTCTACACA‐3′
– m18s reverse 5′‐CGATCCGAGGGCCTCACTA‐3′

### 4.7 Isolation of Single Cells From the CNS

Single cells were isolated from the CNS as follows. Briefly, the mice were anesthetized and euthanized and transcardially perfused with PBS, and the tissues were harvested. CNS tissues were digested with 0.2% collagenase II (Sigma‐Aldrich, St. Louis, MO, USA) for 1 h at 37°C and subsequently separated by gradient centrifugation with 30% Percoll (Cytiva, Little Chalfont, Buckinghamshire, UK) gradient centrifugation for 30 min at 400 *g* at 20°C.

### 4.8 Flow Cytometry (FCM)

Single‐cell suspensions were prepared as described above. For intracellular and intranuclear marker staining, the cells were fixed and permeabilized with fixation/permeabilization solution (BD Biosciences, San Jose, CA, USA) for 30 min at 4°C in the dark, followed by incubation with intracellular or intranuclear antibodies for 30 min at 4°C in the dark. Then, the cells were fixed with 1% paraformaldehyde. Flow cytometric data were obtained with a FACS Verse flow cytometer (BD Biosciences, San Jose, CA, USA) and analyzed via FlowJo software (Version X; Tree Star, Ashland, OR, USA). The antibodies used in the flow cytometry assays were as follows: PE‐conjugated anti‐mouse Nestin (MA5‐23574; Invitrogen, Carlsbad, CA, USA), PE‐conjugated anti‐mouse Sox2 (656,103; BioLegend, San Diego, CA, USA), and PE/Cyanine7‐conjugated anti‐mouse Ki‐67 (652,425; BioLegend, San Diego, CA, USA).

### 4.9 Transmission Electron Microscopic (TEM)

The spinal cord was obtained and fixed separately in 2.5% glutaraldehyde for 2 h, followed by 1% osmium tetroxide for 2 h, dehydrated and embedded in epoxy resin. Sections stained with toluidine blue or uranium acetate and lead citrate were then observed by TEM (Hitachi, Japan). Each myelin sheath was tracked using the ImageJ plugin G‐ratio calculator (G‐ratio = axon diameter/total diameter of myelinated fiber).

### 4.10 Western Blot (WB)

Tissues were homogenized in RIPA lysis buffer (Beyotime, China) supplemented with 1% PMSF (Beyotime, China) and 1% phosphatase inhibitor (Biosharp, China). The lysate concentration was detected by BCA protein quantitative kit (Abbkine, China). The lysates were separated, transferred, and incubated with primary antibody overnight at 4°C and then secondary antibody labeled with horseradish peroxidase for 1 h at room temperature. The protein bands were acquired using Amersham Imager 600 instrument (GE, USA), and analyzed with ImageJ software. The antibodies used were listed as follows: anti‐μ‐opioid receptor antibody (1:1000; ab17934, abcam, UK), anti‐κ‐opioid receptor antibody (1:1000; ab183825, abcam, UK), anti‐POMC antibody (1:1000; SAB2500826, Sigma, USA), anti‐GAPDH antibody (1:2000; 2B5, Abbkine, China).

### 4.11 Statistical Analysis

In this study, all the data are expressed as the means ± SEM. All the statistical analyses were performed with GraphPad Prism software (Version VIII; La Jolla, CA, USA). Statistical analyses were performed using unpaired *t*‐test, one‐way ANOVA, or two‐way ANOVA coupled with Tukey post hoc test. *p* values < 0.05 were considered significant. In all performed experiments, the *n* value represents the number of independently conducted experiments.

## Author Contributions

Conceptualization, Y.L.; methodology, Y.W., X.M., Z.Q., W.Z., X.Z., and J.L.; investigation, Y.W., X.M., Z.Q., W.Z., A.L., C.W., and J.J.; writing – original draft, Y.W.; writing – review and editing, X.X., J.W., Y.M., W.H., S.Z., X.L., H.L., and B.S.; funding acquisition, X.L. and Y.L. All the authors commented on previous versions of the manuscript. All the authors read and approved the final manuscript.

## Funding

This study was supported by grants from the National Natural Science Foundation of China (81774024), the Natural Science Foundation of Heilongjiang Province of China (LH2022H004), and the Special Project of Traditional Chinese Medicine in Heilongjiang Province of China (ZYW2023‐132).

## Disclosure

We did not use any Artificial Intelligence Generated Content (AIGC) tools.

## Ethics Statement

This study did not involve human participants. All experimental procedures involving animals were conducted in accordance with institutional guidelines and approved by the Ethics Committee of Harbin Medical University.

## Consent

The authors have nothing to report.

## Conflicts of Interest

The authors declare no conflicts of interest.

## Data Availability

The data that support the findings of this study are available from the corresponding author upon reasonable request.
